# Health, human rights, and the conduct of clinical research within oppressed populations

**DOI:** 10.1186/1744-8603-3-10

**Published:** 2007-11-08

**Authors:** Edward J Mills, Sonal Singh

**Affiliations:** 1 Programme in International Human Rights Law, University of Oxford, Oxford, UK; 2 Department of Pharmacy, Rhodes University, Grahamstown, Eastern Cape, South Africa; 3 Bloomberg School of Public Health, Johns Hopkins University, Baltimore, USA; 4 School of Medicine, Wake Forest University, Winston-Salem, USA

## Abstract

**Background:**

Clinical trials evaluating interventions for infectious diseases require enrolling participants that are vulnerable to infection. As clinical trials are conducted in increasingly vulnerable populations, issues of protection of these populations become challenging. In settings where populations are forseeably oppressed, the conduct of research requires considerations that go beyond common ethical concerns and into issues of international human rights law.

**Discussion:**

Using examples of HIV prevention trials in Thailand, hepatitis-E prevention trials in Nepal and malaria therapeutic trials in Burma (Myanmar), we address the inadequacies of current ethical guidelines when conducting research within oppressed populations. We review existing legislature in the United States and United Kingdom that may be used against foreign investigators if trial hardships exist. We conclude by making considerations for research conducted within oppressed populations.

## Background

There is an overarching assumption that medical research, in general, is a good thing. Even those who don't subscribe to this arguably simplistic view would hold that medical research is absolutely necessary. This situation is made complex by a widespread expectation that science and technology are expected to be capable of curing all conditions. Society and the medical community have come to the conclusion that research on humans is both necessary and desirable. This poses urgent questions, however, when we consider the poor quality and poor outcomes of most clinical trials and basic science experiments [1-7] As clinical trials and sentinel data expand in number and location, the necessity and relevance of research's worth to participants represents both an ethical and human rights concern [8]. The recent globalization of international clinical trials highlights relatively new questions as to whether conducting research in marginalized or oppressed populations can or should be acceptable.

The number and breadth of international and regional instruments displays the lack of clarity that exists within the biomedical fields of research. Declarations, by their very nature, are non-binding instruments that guide the conduct of research, but may hold mandatory rules within single institutions, not inter-state. Some have asserted that part of the problem is not that there is too little international standard setting but that there is too much of it [9]. We believe that issues arising in clinical trials are not limited to ethical concerns, but at times enter the world of international human rights law. We recognize the need for further discussion and the difficulties of conducting international research and the complexities of contemporary medical research in the developing world, particularly research amongst populations that are known to suffer systematic human rights violations- from here on called 'oppressed'. We recognize that in-light of existing cases of participation in rights and ethics violations, any new declarations or legal instruments must have the requisite teeth to bite when transnational companies and sovereign states engage in questionable foreign research with foreseeable negative consequences.

We have previously addressed the role of researchers in promoting vulnerable population participant rights in respect to HIV clinical trials [10]. Here, we present an overview of considerations for the conduct of research, whether clinical trials or sentinel data collection, when working with systematically oppressed populations. Using case-examples from Thailand, Nepal, and Burma (Myanmar), we recommend considerations regarding the protection of participants and local colleagues in settings with oppressed populations.

### Thailand

Conducting trials within a population that is not just marginalized but is actually under direct threat from government action poses its own problems for researchers. The situation concerning those involved in the drug trade in Thailand remains of grave concern. During Thailand's 2003 'war on drugs,' over 2,500 alleged drug traffickers and users were extrajudicially killed [11]. The former Prime Minister, Thaksin Shinawatra (since ousted in a bloodless coup), publicly denigrated non-government organizations for reporting the human rights abuses internationally [12].

Given these conditions, conducting research that collaborates with Thai authorities and addresses drug user issues creates dilemmas that straddle ethics and international human rights law. Could participation in a state-authorized clinical trial of HIV prevention 'out' an intravenous drug user and potentially expose them to a risk of state-sanctioned violations? In these circumstances, what duties regarding privacy and confidentiality of information exist on the part of the researcher? Although medical records in a clinical trial are confidential, the security of the records is a serious concern, given the state history of behavior amongst this population. It is not unreasonable to suggest that lists of populations can be used to devastating effects when we consider the state-led violence in Rwanda's genocide, Chile's military rule, or China's crack-down on Falun Gong membership [13,14].

A clinical trial that is currently being conducted in Bangkok amongst IDUs displays the complexity and challenges that exists when one examines clinical trials only as an ethics dilemma or only as a legal dilemma [10]. The trial, assessing the effectiveness and safety of oral tenofovir for HIV prevention in 1,600 IDU participants from the Bangkok area, is funded by the US Centers for Disease Control and prevention (CDC) and conducted by Thai research partners. This trial, like others around the world assessing tenofovir, has come under scrutiny for highlighting ethical questions rarely addressed. In this case, the study is funded by a US state transnational organization. US state funds are not permitted to cover the supply of drug paraphernalia and as a result, clean needles are not provided to participants. However, article 29 of the Helsinki Declaration states that '*benefits, risks, burdens and effectiveness of a new method should be tested against those of the best current prophylactic *'interventions [15]. Further, article 23 of the recent UNGASS Declaration of recommitment to HIV/AIDS, signed by both Thailand and USA [16], notes the affirmation of access to essential commodities, including sterile injection equipment and harm-reduction efforts related to drug use. This raises important legal and ethical concerns. Domestic laws, such as the US policy, are countered by international norms for provision of clean needles [16]. Should a foreign state's (US) laws override an internationally agreed upon standard of clinical trials, the Helsinki Declaration or the UNGASS Declaration, signed by both the Thai and United States Medical Associations?

### Nepal

In autumn 1995, the United States Armed Forces Research Institute of Medical Sciences established the Walter Reed/AFRIMS Research Unit-Nepal field unit in Kathmandu, Nepal, for the study of Hepatitis E, a water-borne disease with a high maternal mortality rate.

In late 1999, plans for a Phase II/III clinical trial of a Hepatitis E vaccine were approved by the Nepal Health Research Council and the U.S. Army's Human Subjects Research Review Board[17]. The vaccine had been patented by California based biotech company Genelabs, and licensed by SmithKline Beecham (now GlaxoSmithKline). It was subsequently announced that the trial would begin in mid February 2000 with the screening of 8,000 volunteers in Lalitpur, 3,000 of whom would be enrolled in the trial receiving the vaccine or placebo. Following the trial's announcement Genelabs' share price nearly doubled, reaching their highest level in the nine year history of the company [18]. While shareholders were celebrating their good fortune, articles began to appear in Nepali newspapers suggesting that the Nepali population was being taken advantage of by Western pharmaceutical companies. Concerned about the controversy and possible political fall-out, local government officials from Lalitpur met to discuss the trial. They concluded that the trial should not continue as scheduled until the researchers satisfied the governing committee that relevant actions such as post-trial access had been accomplished.

During this intermission, the controversy heightened, with critics asking whether there had been adequate education to allow informed consent among the trial participants and why researchers would not contribute to the clean up the water supply – Thailand had been initially planned as trial site but the disease had been largely eradicated in the 80's and 90's due to advances in water sanitation. The Deputy Mayor had asked for a hospital to be built, as proposed profit sharing was considered out of the question. The prohibition against the trial was upheld and it was moved to a community that predictably did not raise any objections: recruits from the Royal Nepalese Army.

As with any army population, the use of serving members of the military as trial participants is dubious at best since questions of informed consent and undue influence must be asked in light of what is necessarily a highly ordered and authoritative community, even in developed nations [19]. Upon entering military service, soldiers necessarily give up certain rights enjoyed by private citizens and it is conceded that if a military is to be at all effective there must be some element of the destruction of individual autonomy. However, can there consistent informed consent in the RNA when there is only 44% literacy rate? [18] Nepal's military is also well known for torture, disappearances and inadequate and infrequent pay for its members [20]. Can conscripts ever be said to engage in any military activity voluntarily? How much coercion is involved by decision-makers? Can a participant withdraw? [21] When Sachit Rana, Chief of Army Staff at the RNA, claims "the army can do anything it wants – shoot at people, use bombs, arrest people" [22], it is difficult to imagine a more unsuitable population for such a trial. Surely institutions that are guilty of mass human rights abuses, such as the RNA or the Thai drug authorities, should be neither subjects nor partners for human subject research, yet in this case they were both – a sad state of affairs seeing as the trial had been approved by the National Institute of Allergy and Infectious Diseases and US Army IRBs.

### Burma (Myanmar)

In the examples of Thailand and Nepal, we present cases of clinical research that could have been conducted in populations other than the oppressed participant populations. However, should research ever be conducted in populations with foreseeable oppression?

There are few countries in the world that can boast of such egregious human rights violations as Burma (Myanmar). A military government, since 1958, has ruled Burma. In 1988, a new military government, the State Law and Order Restoration Council (SLORC) took over and renamed the country Myanmar. SLORC would change Burma's name to Myanmar and impose martial law, repressing any democracy movements [23]. SLORC would begin a systematic train of human rights abuses including forced labour, rape, torture and murder [24]. Burma's present authoritarian military government, the State Peace and Development Council (SPDC), continues to operate a strict police state and drastically restricts basic rights and freedoms. Its military is estimated at 350,000 and as many as 70,000 are estimated to be child soldiers [25,26]. Freedom of expression, assembly, and association are not respected and the arbitrary detention and incarceration of those expressing their political opinions continues [26]. As a result of the military violations, the United Nations appointed a country specific Special Rapporteur on the situation of human rights Myanmar since 1992. Most recently, the *Doe *versus *Unocal *case set important precedent [23]. This case, settled in 2005, addressed whether Unocal, a multinational oil company, could be held responsible for crimes against humanity committed by the Myanmar military, a business partner. The case was found admissible and demonstrates that multinational companies that partner with repressive states may be responsible for their partnering states actions. Given the widespread nature of the human rights violations, it is difficult to determine whether researchers should choose to conduct their work in such an atmosphere. We recognize that even in vulnerable populations, relevant research is necessary if the population has exception medical needs, as in the case of malaria, which is often geographically distinct in terms of drug-resistance and endemicity [27]. This may explain why so many malaria trials have been conducted in Burma.

When dealing with geographically specific diseases, we need to consider the importance of the research initiatives. Trials evaluating artesunate treatment for malaria have been successfully conducted in Burma amongst military recruits and their families. These clinical trials, approved by the University of Oxford ethics review committee in the UK, display the inadequacy of our current ethics frameworks. Regardless of actual human rights violations within the trial, it seems implausible that the ethics review committee members from Oxford have a through knowledge of the situation for Burmese recruits residing in Burma. The Oxford review committee insisted on a partnership between the Oxford researchers and the SPDC Ministry of Health (MOH). At this time it is worth noting the debacle last year between the SPDC and the Global Fund to Fight AIDS, TB, and Malaria resulted in the withdrawal of funding for HIV/AIDS programmes as the junta had prevented allocation of resources to the required programmes and suspected of using the money for military purposes instead [28].

Given the realities of research infrastructure, clinical trials conducted in Burma must partner with the relevant local authorities. These trials raise important and difficult questions. As illustrated in the Doe vs Unocal case, partnering with the Burmese government is against many state laws and may implicate a research body in crimes. We would argue that partnering with such an organization may be excusable in limited circumstances – where there are compelling and pressing reasons why such research cannot be conducted amongst another population. Indeed, several trials demonstrating the effectiveness of artesunate based therapies for severe malaria have instigated a national policy change regarding treatment within Burma, demonstrating that this research was necessary and in certain circumstances can result in important improvements for the populations at risk [29].

## Discussion

These case studies illustrate the complexities and potential pitfalls of examining research from simply an ethical or simply a human rights legal framework. As previously stated, there are many international instruments that confer and safeguard the rights of participants in clinical trials. The first widely-adopted instrument was the Nuremburg Code; followed in 1964 by the Helsinki Declaration (since revised 7 times by the World Medical Association) [15]; the 1966 International Covenant on Civil and Political Rights (CCPR)(particularly Article 7 as it relates to consent for medical and scientific experiments) [30]; the Council for International Organizations of Medical Sciences (CIOMS) international ethical guidelines in 1993 (since revised) [31]; and, the 1996 International Conference on Harmonization of Technical Requirements for Registration of Pharmaceuticals for Human Use – Good Clinical Practice: Consolidated Guidelines [32]. On a European level [EU], the EU has issued its directive on good practice in clinical trials [33] and the Council of Europe has issued a Convention on Human Rights and Biomedicine on biomedical research [34]. Most recently, UNESCO has developed a Universal Declaration on Bioethics and Human Rights [35]. Although there is considerable overlap between these documents there are also dramatic differences between them, that no doubt contribute to general confusion and the possible conflation of discrete legal principles that has resulted in some pausing to question whether they can contribute to the development of a comprehensive international framework [9].

Given their brevity, the Nuremberg Code and the CCPR are of minimal benefit today in any discussion on human rights in clinical trials. Neither document recognizes the distinction between therapeutic and non-therapeutic research. This fundamental deficiency means that they have largely been ignored by the medical profession. A logical consequence of a strict construction of Article 1 of the Code and Article 7 of CCPR (both addressing consent) would mean that consent would be necessary in all circumstances: those who become unconscious due to an accident or disease or those who are mentally handicapped could not, if no standard treatment exists, be offered new therapeutic measures that might restore their health or save their lives. Such a rigid interpretation would mean that the respective provisions exclude many of those they were designed to protect. Furthermore, in the case of Article 7 CCPR it is obvious that the provision is to be read as a whole and that the caveat contained in the second sentence must be read in conjunction with the first.

"No one shall be subjected to torture or to cruel, inhuman or degrading treatment or punishment. In particular, no one shall be subjected without his free consent to medical or scientific experimentation."

The Article prohibits experiments that violate the integrity of the person by cruel, undignified or inhuman treatment. Clinical research carried out in accordance with general principles would not violate this provision. Yet the right to health argument confounds this. At the recent XVI International AIDS Conference in Toronto, UNAIDS presented an ethical argument as to why trial participants that seroconvert during a trial do not require access to effective treatment [36]. UNAIDS representatives considered these ethical and not obligatory legal issues. We were dumbfounded to see a UN agency using an ethical argument to abstain from providing the highest attainable standard of health, a human right guaranteed in so many UN legally binding documents.

Although the ethics declarations themselves are not binding legal documents, they arguably represent customary law – law based on an established pattern of behaviour that can be objectively verified within a specific field. The Declaration of Helsinki is a respected document of long standing and esteemed pedigree that is cited in numerous international and national legal instruments, giving it force of law in certain jurisdictions and under certain well-established principles, such as voluntary participation and informed consent [37]. It is signed by all member-state medical associations. It is also subject to intermittent revisions approved by the General Assembly of the World Medical Association (WMA), and therein lies its vulnerability: the WMA is a non-governmental association and is subject only to private law while the rights of participants in clinical trials is increasingly being viewed through the prism of international human rights law [37]. There is little recourse for most Nepali rural dwellers or drug users who might have participated in a trial that conceivably constitutes a violation of Article 19 of the Declaration (access to effective interventions) or the RNA conscript whose participation is, on first appearance, a violation of Article 20 (informed and voluntary consent) if there has not been ratification by their respective national legislatures.

This shortcoming is even more pronounced with respect to the CIOMS since the method by which proposals are adopted is much less transparent. It was the covert nature of the deliberations that led some to criticize the 1993 version of the guidelines claiming that protection had been over emphasized and more pragmatism had been called for, reflecting much more the interests of the research community than the basic rights of the individual or official WHO policy [38]. However, the 2002 CIOMS guidelines do make reference to the Declaration of Helsinki and indeed claim it is "*the fundamental document in the field of ethics in biomedical research*."[31] The updated version also enunciates the laudable goal that "*the research is responsive to the health needs and the priorities of the population or community in which it is to be carried out; and any intervention or product developed, or knowledge generated, will be made reasonably available for the benefit of that population or community*."[39]' Nevertheless, as stated in the Declaration of Helsinki, human rights issues are coming to be seen as within the proper domain of public law and private law remedies such as negligence and the tort of trespass do not constitute a potent deterrent for unscrupulous researchers operating in under-developed countries where access to legal advice is scarce and/or prohibitively expensive. But if the long arm of civil law can reach them in their largest market there may be light at the end of the tunnel.

### Alien Tort Claims Act

One potentially powerful tool in the rebalancing of rights within clinical trials in vulnerable populations is the United States' Alien Tort Claims Act [ACTA, 28 U.S.C., 1350] [40]. Considering that the majority of clinical trials conducted in developing nations are supported by US based organizations [5,41], the ATCA is particularly relevant. It was originally part of the Judiciary Act of 1789 and was one of the first laws of the new American Republic. A federal law, it allows lower courts to hear cases in violation of international law or the "*law of nations*". There have been 3 modifications since its passing and now reads: "*The District Courts shall have original jurisdiction of any civil action by an alien for a tort only, committed in violation of the law of nations or a treaty of the United States*." The statute had been invoked only five times in the first 200 years of its existence and was arguably a rather ineffective remedy [42]. There is renewed interest in the use of the tort as a tool for human rights litigation. One of the most important ATCA decisions, in 1980, the Second Circuit court addressing *Filartiga v. Pena-Irala *[43], held that the ATCA represented liability for torture committed using state authority as it found a Paraguayan police official that visited the US to be criminally responsible for his actions in torturing and disappearing a young man in Paraguay. Torture, in this case, was viewed as illegal under any international circumstances as no reasonable state should permit torture. In 1995, the tort was used to serve Radovan Karadzic for atrocities in Bosnia in the *Kadic v. Karadzic *[44] case, that expanded the statute's reach to private actors. These decisions put to rest (at least on the Second Circuit) questions regarding whether or not the Act conferred mere jurisdiction or an actual substantive cause of action [45]. It should be noted, however, that ATCA does not grant the federal courts jurisdiction over foreign governments or their agencies [46]. Nevertheless, it is clear now from a developing body of case law, and the recent case of *Doe I v. Unocal Corp*, a case that identified Unocal Oil Corporation responsible for atrocities committed in partnership with the Burmese junta, that the liability of multinational corporations falls squarely within the domain of ACTA. Most recently, in 2007 US Federal Prosecutors used the ATCA successfully against Chiquita Brands International for their funding of Colombian rebel groups resulting in fines [47].

For a claim to be successful under ATCA there are three requirements: (i) there must be alien plaintiffs; (ii) suing for a tort; (iii) committed in violation of international law [24]. The first two required elements are relatively straightforward; however, the third presents difficulties as it requires American courts to consider customary international law or treaties and attempt to give effect to customary law, where actual law had not previously existed. This task is simplified somewhat if the alleged violations concern international laws that are *jus cogens *– a violation of international norms from which no derogations are permitted. The *Unocal *case concerned forced labour which was likened to slavery – a blatant *jus cogens *violation. However, the court held that "a *jus cogens *violation is sufficient, but not necessary, to state a claim under the ATCA" [23]. With this in mind there is an argument to be made that a violation of a provision of the Helsinki Declaration resulting in foreseeable injury could found a claim under ATCA. Might a conscript in the Burmese Army or the Royal Nepali Army file a suit against SEAQUAMAT or GlaxoSmithKline respectively for their part in a trial alleging a lack of informed consent and therefore an infringement of Article 20 of the Declaration of Helsinki? Could the family member of a Thai IDU who was subject to extrajudicial execution sue a pharmaceutical corporation for retaining a Thai police officer as security by alleging a violation of the victim's right to confidentiality as expressed by Article 21?

These questions have recently been answered, to some extent, when the State of New York Federal Court permitted a Nigerian claim against a multinational pharmaceutical company, Pfizer Ltd, for the conduct of therapeutic research using an experimental antibiotic, Trovan, versus the on children without receiving informed consent during a meningitis epidemic in Nigeria [48]. In this clinical trial (n = 200), 11 children died and others were left blind or paralyzed. This case was dismissed from the US courts in 2002, finding on the grounds of that the case be completed in Nigeria, where able courts exist (*forum non conveniens*), but did identify Pfizer US as a state-actor, given its financial inter-dependence with the US government, and the approval of the US Food and Drug Administration for the trial. In Nigeria, criminal charges have been brought against Pfizer officials and a civil suit seeking billions in damages has been sought. The case is ongoing.

On June 29, 2004, in the case of Soza vs *Alvarez-Machain *[49], the US Supreme Court upheld the ATCA in recognition of serious human rights abuses. The court did however, limit these abuses to "specific, universal, and obligatory international norms (violations of safe conduct, infringement of the rights of ambassadors)." Nevertheless, courts may not be slow to accept new causes of action under ATCA as recent decisions on the concept of complicity and secondary liability differ. ATCA claims are generally held against private organizations that may be working independently or as state-actors. However, it cannot be applied to the state itself. In the Thai and Nepali trial examples, the trials were funded by the US state, with some involvement however peripherally with the drug manufacturer. The New York District Court has held that liability in international law "for knowing practical assistance or encouragement which has a substantial effect on the perpetration of the crime" is a core principle that forms the foundation of customary international legal norms [50]. Only time will tell which approach is ultimately adopted.

The arguments laid out towards the use of the ATCA and United Nations Global Compact [51] on good corporate responsibility are limited as they apply predominantly to multinational corporations and not to states. However, states are the largest funders of clinical research on neglected diseases in developing settings and in all case examples that we apply here, states have either funded or coordinated the clinical research. There are state laws regarding state behaviour when dealing with oppressive regimes. Without being extensive, we briefly describe relevant laws in the United States and the United Kingdom.

### 502B

The US government has issued communications to sitting judges claiming that ATCA should be given a narrow interpretation, arguing in support of limiting the role of ATCA to finding corporations liable for human rights violations. Such conduct should be seen in light of existing legislation designed to prevent the government from trading with known human rights abusers. Section 502B of the Foreign Assistance Act of 1961 (now coded as 22 U.S.C., 2304) as amended reads "...*a principal goal of the foreign policy of the United States is to promote the increased observance of internationally recognized human rights by all countries*." The law enforces that no state-funds made available by the Foreign Assistance Act should be dispensed to foreign military units with known human rights violations for weapons or training. In the spirit of the law, the provision of funding to Thai military or Nepali military seems specious. Although the statute addressing defense appropriations allows for derogation if such action is certified by the Secretary of State owing to "extraordinary circumstances", it is difficult to see why the jurisdiction of the US courts should be denied to victims of a corporate-foreign-regime-relationship when such a regime would not even qualify for US foreign aid by virtue of them being known human rights violators. This law, commonly known as the Leahy Law (after Senator Patrick Leahy) has most famously been implemented with regards to assisting specific units in Colombia's military, which was circumvented by units swapping out abusers to make specific units clear of abusers.

### United Kingdom

There is not one exhaustive statute that deals with preventing government and the private sector partnering with rogue states or abusers of human rights. The UK has passed a number of statutes that authorize the imposition of trade or economic sanctions, providing some inference that illegal behaviour outside the UK is viewed with condemnation.

### The United Nations Act 1946

This Act is essentially an enabling statute that brings UN Security Council resolutions into the law of England and Wales. Once again, under Chapter VII (Article 41) of the United Nations Charter, if the UN Security Council determines that a threat to the peace, a breach of the peace or an act of aggression has occurred, it may decide what measures shall be taken to maintain or restore international peace and security. A UN Security Council decision taken pursuant to Article 41 imposes a legal obligation on the UK (as a UN Member) to take all necessary measures to give effect to the decision domestically. When a resolution imposes sanctions, the UK must introduce them into domestic law. This is done by passing statutory instruments under the United Nations Act.

Section 1(1) of the Act enables Parliament to pass legislation in furtherance of Article 41. It provides:

"If...the Security Council of the United Nations call upon His Majesty's Government in the United Kingdom to apply any measures to give effect to any decision of that Council, His Majesty may by Order in Council make such provision as appears to Him necessary or expedient for enabling those measures to be effectively applied..."

The UN Security Council resolution will be enabled in domestic law by way of an Order in Council (a statutory instrument) made by the Foreign Secretary, and laid before Parliament. Such an instrument will have force of law and a violation of it could bring a term of imprisonment of up to 6 years.

Recent example of Orders in Council made under the Act include: The Sudan (United Nations Measures) Order 2006 [52]; The Lebanon and Syria (United Nations Measures) Order 2005 [53]; The Democratic Republic of the Congo (United Nations Measures) Order 2005 [54].

These Orders in Council are simply a mechanism by which UN Security Council resolutions are enacted and made enforceable in the UK under domestic law. They do not proscribe trade or aid with nations or organisations who are not subject to a Security Council sanction and who may be gross human rights violators. Further, they do not forbid all trade even with proscribed countries but only those items specified in the Security Council resolution (most often arms, aircraft, etc.).

### The Export Control Act 2002

The *Import, Export and Customs Powers (Defence) Act 1939 *provided the Secretary of State with a general power to impose import and export controls on goods. The 1939 Act was considered to be a temporary measure to deal with the emergencies of the time. However, the 1939 Act has remained in force (although it was amended by the Import and Export Control Act 1990 to allow it to continue in force without relying on the continued existence of "the emergency" that existed in 1939).

The Scott Inquiry into *Export of Defence Equipment and Dual-Use Goods to Iraq and Related Prosecutions *in February 1996 identified a number of limitations in the 1939 Act, including the lack of parliamentary scrutiny of secondary legislation made under the Act and the absence of any indication of the purposes for which export controls may be imposed. The 2002 Act was passed after a government white paper was circulated that took account of some of the concerns raised by Lord Scott.

In the absence of a UN Security Council resolution embargoing trade with a particular group or nation, the UK has the power to prohibit such trade by use of Orders in Council made under the *Export Control Act 2002*. But are clinical trials a trade issue? Section 5(2) of the Act states that " [c]ontrols of any kind may be imposed for the purpose of giving effect to any Community provision or other international obligation of the United Kingdom." This refers to bilateral and multilateral agreements which the UK may have, notwithstanding their UN obligations. In practice it is most common for Orders in Council made under the Act to be in furtherance of European Union objectives, an example being under Title V of the Treaty on European Union (provisions on a common foreign and security policy).

Although there is guidance from the EU on trading and engaging with rogue states (for instance the European Union Code of Conduct on Arms Exports) the regulations are not all-encompassing. Matters of defence and foreign policy are still within the proper domain of member states and since both of these areas are governed to a large extent in the UK by the Royal Prerogative it is not difficult to see instances where partnering with questionable regimes would be possible since it had not been specifically proscribed by the EU.

The power of US 502B is that it requires the Secretary of State to justify why trade/aid should be allowed with such a nation despite its hideous human rights record. Whether or not 502B has had a positive impact on foreign trade and the ability to partner with rogue regimes is difficult to say, since exemptions can be made under it if they are certified by the Secretary of State. Although this is not an ideal safeguard, it does force politicians to publicly declare the reasons for partnering with such a regime.

### Considerations for the Conduct of Research within Oppressed Populations

As the costs of research increase it is no wonder that pharmaceutical companies are looking to developing nations to conduct much of their research. Much of the marketing of multinationals is now trumpeting their commitment to human rights and the environment. Indeed many of the world's largest companies have apparently embraced guidelines drawn up by the United Nations concerning ethical conduct, such as the UN Global Compact and the UN Norms on the Responsibilities of Transnational Corporations and Other Business Enterprises with Regard to Human Rights [55]. Yet in the same breath these companies are lobbying in Washington in an attempt to have the Alien Tort Claims Act repealed by propagating the fiction that it will make it impossible for well-intentioned companies to know what conduct might subject them to liability or that it will stifle foreign investment. It is not cynical to suggest that this is the case because the Global Compact and the UN Norms do not contain a word about enforcement.

It is evident that in the absence of any effective international legal framework to address the dilemmas and complexities of research in vulnerable populations we are set to see the further abuse of human rights. Table 1 lays out necessary considerations for the conduct of clinical research in oppressed populations. It must be the case that if companies or states are going to reap the benefits of conducting research on vulnerable groups they carry out they requisite appropriate consultation. Ethical review boards cannot be a 'one size fits all' but must take into account local concerns and address those concerns in liaison with local representatives and further in-roads must be made into the entrenched hostility on the part of drug companies to post-trial medical care. The greater the atmosphere of vulnerability the greater the level of consultation must be; in settings with foreseeable human rights violations, such violations much be captured. The time is ripe for an international legal instrument to address these concerns and upon ratification in national legislatures to provide a substantive cause of action without excessive restrictions on *locus standi *which result in companies never being brought to book as 'victims' never make it to court. These are complex issues that demand attention, and in the absence of an international convention, it falls to creative and bold jurists to push for these issues in courts of law and various international fora.

**Table 1:** Considerations for conducting research in oppressed populations

**Health Promoting**	For clinical effectiveness interventions, interventions should be health promoting and address a clearly population relevant illness. There should be a clear plan for how research findings will assist community, based on *a priori *agreement on how findings will be used. This requires active engagement with target groups and plans for how findings should be interpreted with target group members.
**Opportunities to avoid exposures should be afforded to participants**	Exposures may include exposures to the target diseases or to human rights violations. This should include education (e.g. condom use, needle provision and needle cleaning); assistance with known human rights promoting activities or possible escape; and, the avoidance of remaining in exposed status.
**Planned efforts to determine the human rights status of a particular population**	This may include formative research and background reviews of human rights NGO literature.
**Community participation commensurate with level of political oppression**	There are few reliable ways to measure oppression. Due to the large geographical and cultural heterogeneity, there may not be a unified voice and so we need minimum standards for Community Advisory Boards (CABs).
**CABs must include mixed levels of education, oppressive situations, age and gender**	They should be able to inform on the importance of the research and therefore, acceptability. CABs may need to be outside target areas (i.e. Burma)
**The more oppression, the more reason for research required**	Assuming researchers cannot improve oppressive setting, the conducting research may be placing participants or colleagues at risks. Oppressive conditions may make population 'captive'
**HR violations should be captured**	Good research captures all health outcomes determined of interest in clinical event forms. In certain conditions we can anticipate HR violations. Case report forms should inquire of HR violations. This may allow for subgroup analyses.
**Report an Ethical methodology**	Ethics and human rights issues are not black or white issue (ethical or not ethical), but require explanation to determine challenges that exist in the research settings. It may well be appropriate to advocate for a limitations section of the article that addresses what could not be accomplished. This could be advocated by the Consolidated Standards for Reporting of Randomized Trials Group (CONSORT), a group that advocates minimum standards of reporting clinical research. It is only through acknowledgement of these issues that the field can be advanced.
**Measure long-term impact, for informing "customary research"**	We need to determine if the research has improved conditions, or worsened it? Has 'Western' research improved or reduced the quality/expectations of indigenous research? Finally, we need to determine if post-trial access was accomplished and what care was provided for post-trial injuries related to participation.

Table 1 highlights issues for consideration of the conduct of oppressed populations. We have previously listed considerations for conduct in difficult to protect populations, and these previous recommendations are updated here. These recommendations recognize that research is often required within specific populations, but that research should be health promoting, rather than risking placing specific or large groups of a population at risk of violence, detention, or other state-led abuses. While no list is exhaustive and each setting is likely to be substantially different, these considerations address specific issues that are applicable to oppressed populations, where the likelihood of harm is impending. We predominantly argue that where oppression exists, the argument for conducting research in that population needs to substantially outweigh the negatives of not conducting the research. The central argument is the optimal balance between ensuring the safety of participants while advancing the needs of science. The "intentionality" of the research endeavour should not only be to advance scientific knowledge but also offer practical benefits, or at least the hope of future benefits for the oppressed population. Researchers need to bear the burden of proof and strive to minimize coercion. They need to identify representative leaders who represent the diversity of opinions, conduct public deliberations in the spirit of participatory decision-making and try to ensure decision making as much as possible at the local level. Some authors argue for a " modus vivendi " approach that favours decision-making via compromise rather than rational consensus. As we alluded to at the beginning of this paper, the assumption that research will necessarily lead to population benefits is naïve and is not supported by evidence. Good intentions alone are likely to be insufficient in protecting potential participants and so extra caution and planning is required.

## Conclusion

As the medical research world becomes increasing globalized, there is a need to make research both methodologically valid and culturally valid. In the examples that we have highlighted, conducting research within oppressed populations stretches the current norms of medical ethics as well as stretching the current capabilities of international law. To rely simply upon minimum standards of non-binding and vague medical ethics instruments for conducting research within these populations is both naïve and culturally insensitive. Human lives are inherently complex and no single ethical framework, including ours can claim to capture the complexity of research and understand the ethical dilemmas that arise in these diverse settings. Paradoxically, legal course is least available to oppressed populations where the need is greatest. Ethical principals and legal approaches outlined above are not intended to be rigid inflexible pillars, rather as signposts along the way for researchers interested in grappling with this challenge of conducting research within vulnerable populations. In accordance with universal principals of justice, the "effective " participation of oppressed populations in decision-making will be an instrumental step in combating the social, economic and political forces of globalization that constrain human capabilities.
